# Selective Complex Precipitation for Ferro-Chrome Separation From Electroplating Sludge Leaching Solution

**DOI:** 10.3389/fchem.2021.592407

**Published:** 2021-06-16

**Authors:** Li Jinhui, Wang Ying, Wang Yudong, Gao Yang, Yang Yang, Wang Ruixiang

**Affiliations:** School of Metallurgy and Chemical Engineering, Jiangxi University of Science and Technology, Ganzhou, China

**Keywords:** electroplating sludge, the separation of ferrochromium, complex precipitation, selective, transformation

## Abstract

In this paper, aiming at the problem of chrome-iron separation in electroplating sludge, the separation of ferrochrome by complexation and precipitation with benzoic acid as complexing agent is achieved. The optimal conditions consisted of a 1: 3 molar ratio of Fe^3+^: C_6_H_5_COOH, a reaction temperature of 30°C, a final pH of 2.5 and a reaction time of 2 min. The separation rate of the iron was 97.38% and the rate of loss of chromium was only 3.59%. The ferrochromium separation products were analyzed by XRD, fluorescence spectroscopy, infrared spectroscopy and H NMR Spectroscopy in order to study the mechanism of precipitation. The results showed that benzoic acid preferentially forms a complex with iron and iron benzoate precipitates with an increase pH. The iron benzoate crystals have a fine particle size, settle rapidly and are easy to filter. The separation of Cr ^3+^/Fe^3+^ was successful using our methodology.

## Introduction

The electroplating industry in China produces approximately 10 million tons of electroplating sludge every year, which is classified as hazardous waste due to containing harmful substances, for example, chromium (Wei-hua and Xue-fu, 2006; Qiu-yue, 2014). On the other hand, the chromium present in the electroplating sludge is an important resource in the stainless steel and electroplating industry. Moreover, China is short of chromium resources and needs to import a large amount of chromium every year. Therefore, the recycling of valuable metals from electroplating sludge can not only help resolve the issue of environmental pollution but also promote the reutilization of waste resources (Chen et al., 2005; Zhang et al., 2005; Li et al., 2013; Liqing et al., 2019). At present, common methods for the utilization of chromium-containing electroplating sludge mainly includes acid leaching, ammonia leaching, electrodeposition, microbial metallurgy, and material utilization among others (Jin-ping et al., 1996; Yi et al., 1999; Fu de, 2002; Silva J. E. et al., 2005; Silva J. et al., 2005; Chen Ke, 2007; Cheng et al., 2010; Guo et al., 2010; Xian, 2012; Zheng et al., 2014). The most popular method used in industry is acid leaching, step precipitation and solvent extraction. In the hydrometallurgy process, the separation of chrome-iron is a relatively important step because of the similarity between Fe^3+^ and Cr^3+^. When the Fe^3+^ ion is separated via neutralization, the Cr^3+^ ion is usually wrapped around or precipitated as hydroxide with iron hydroxide, which results in the loss of chromium and secondary pollution (Cheng-yan et al., 2008; Yuan et al., 2013). Majone (1986), Hu et al. (2006), Jian-Hui et al. (2011), respectively, used the goethite method, the Mohr salt crystallization method and cupferron as a precipitating agent to separate chromium and iron successfully, but the processes are relatively complicated and the problem of secondary pollution cannot be completely resolved. This prevents these methods from been widely used in industry. Silva et al. (2006) oxidized Cr^3+^ to Cr^6+^ and used the solubility difference between Cr^6+^and Fe^3+^in an alkaline system to achieve separation, however, Cr^6+^is highly toxic. Zheng et al. (2020) used crystal modification to recover Cr from electroplating nano sludge. The process control is complex and the crystallinity control is not easy. kolthoff et al. (2002) used ammonium benzoate to separate chromium and iron can not solve the problem of wastewater discharge of amine, so this method can not be applied. Bewtra et al. (1995) used the adsorption of metal ions by bacteria to treat electroplated sludge, but poor biological selectivity to metal ions of this method limits its application.

In order to realize the recovery of chromium from electroplating sludge, an efficient procedure for the separation of chrome-iron must be developed. In this study, a new method for the separation of ferrochromium by a selective complexation precipitation is achieved using benzoic acid as complexing agent which can form a insoluble complex with iron in aqueous solution. The results of our study provide a new method for the comprehensive recovery of chromium from mixed electroplating sludge.

## Experimental

### Reaction Principle

**FIGURE 1:**
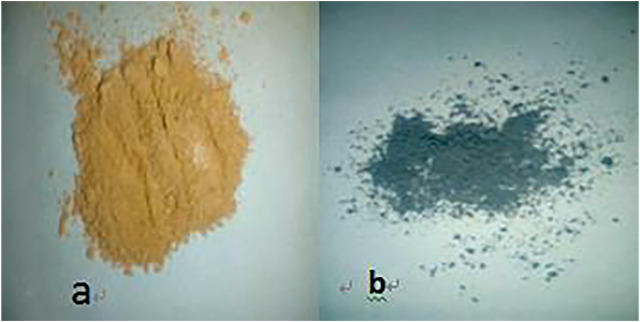
**(A)** Coordination products of benzoic acid and iron ions; **(B)** Coordination products of benzoic acid and chromium ions.

Benzoic acid was found to be the best ligand. Under certain conditions, benzoic acid can coordinate with Fe^3+^ and Cr^3+^ to form stable complexes, which will crystallize and precipitate after saturation. The coordination of Fe^3+^ and Cr^3+^ with benzoic acid in aqueous solution is shown in Eqs. 1, 2. In the experiment, pure Fe^3+^ and Cr^3+^ sulfate solutions with the same concentrations were prepared, and the benzoic acid ethanol solution was slowly added under the action of magnetic stirring in a constant temperature water bath. The products obtained by filtration, separation and drying are shown in Figure 1.

**Figure:**
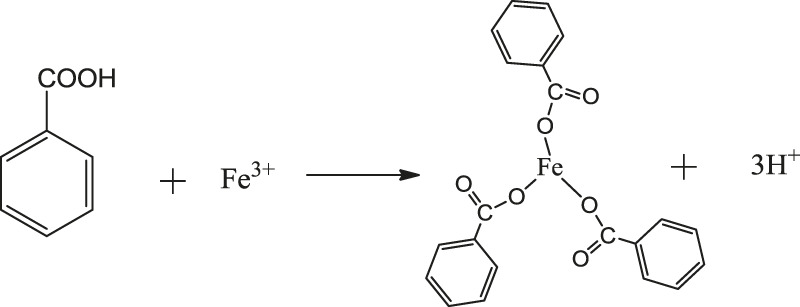


**Figure:**
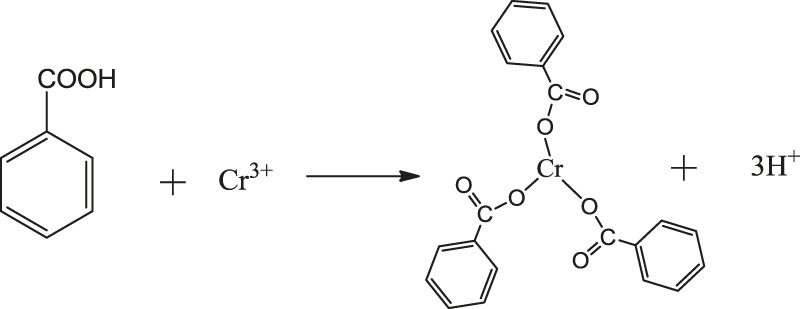


**FIGURE 2:**
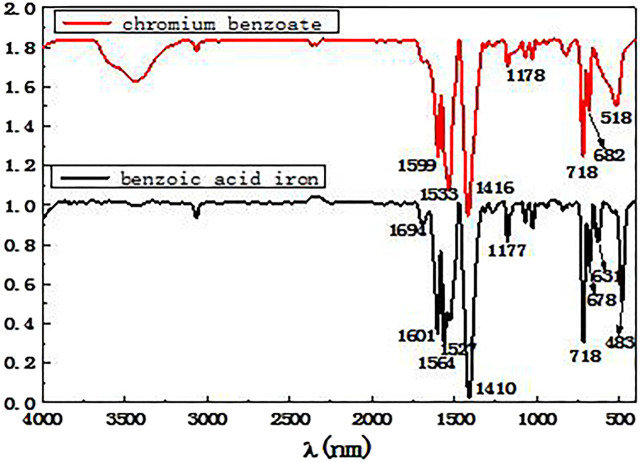
Infrared spectra of chromium benzoate and benzoic acid iron products.

The reaction products of benzoic acid with both iron and chromium were analyzed by infrared spectroscopy, and the resulting infrared spectra are shown in Figure 2.

From the analysis of the spectra 2, it can be seen that the absorption peaks around 1,601 and 1314 cm^−1^ correspond to the stretching vibration absorption bands of the benzene ring carbon-carbon bonds. The peaks at 3,058, 1,601, and 718 cm^−1^ can be ascribed to the three characteristic absorption peaks of the benzene ring. The strong absorption peaks appearing around 1,527 and 1,410 cm^−1^ correspond to the asymmetrical and symmetrical stretching vibration peaks of the carboxylate groups in the benzoate. The above results show that the coordination reaction of benzoic acid with chromium and iron ions produced the corresponding products. The spectrum of the product formed from the reaction of benzoic acid and iron shows significant peaks at 631 and 483 cm^−1^, whereas the spectrum of the benzoic acid and chromium reaction products show broad absorption peaks at 518 cm^−1^ and no peaks at 631 cm^−1^. However, the coordination ability of benzoic acid with different metal ions varies, and conditions such as reaction temperature, solution acidity and basicity must be adjusted in order for the benzoic acid to form stable complexes with Fe^3+^ and Cr^3+^ ions. Thus, benzoic acid was added to a mixed solution of ferrochrome, and an appropriate reaction temperature and reaction time were selected. Subsequently, by adjusting the pH of the solution, the reaction was promoted and benzoic acid was selectively precipitated and separated from the ferrochrome.

### Experiment Concerning the Separation of Ferrochromium by Selective Benzoic Acid Complexation and Precipitation

**FIGURE 3:**
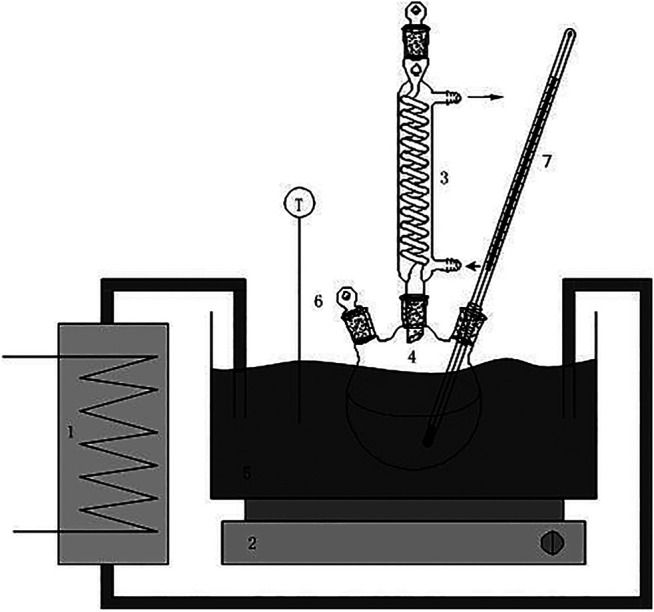
Equipment sketch: 1: temperature-controlled bath with external flow; 2: magnetic stirrer; 3: tap water-cooled condenser; 4: round-bottom flask with three holes; 5: water bath; 6: outlets for batch addition and solution sampling; 7: thermometer.

In the electroplating sludge leaching solution mixed with chromium and iron, the benzoic acid coordination agent which was used was that of an ethanol solution of benzoic acid. The experiment was carried out by stirring at a constant temperature in a water bath. The benzoic acid ethanol solution was slowly added dropwise to the mixed chromium-iron solution, and then the pH was adjusted with ammonia water to achieve selective complexation and precipitation of the iron ions. The effects of reaction time, reaction temperature and benzoic acid dosage on the separation of ferrochromium by the selective benzoic acid complexation and precipitation method were investigated. The experimental setup is shown in Figure 3.

### Analysis Method

In this experiment, o-phenanthroline spectrophotometry was used to determine the Fe^3+^ content in the filtrate after the reaction, and WFX-1380 atomic absorption spectrophotometer was used to determine the Cr^3+^ content in the filtrate after the reaction.

The formula for the calculation of the precipitation rate of the ferrochrome ions in this paper is as follows:

$$\text{Precipitation}\;\;\text{rate}\;=\;\frac{C_{1}\times V_{1}-C_{2}\times V_{2}}{C_{1}\times V_{1}}\times 100\%$$

In the formula, C_1_ is the initial solution concentration (g/L), V_1_ is the initial solution volume (L); C_2_ is the concentration of metal ions (g/L) in the filtrate at constant volume, and V_2_ is the volume of the solution at constant volume of the filtrate (L).

## Results and Discussion

### Separation of Ferrochromium by Precipitation of a Benzoic Acid Complex

#### Effect of Solution pH on Ferrochrome Separation

**FIGURE 4:**
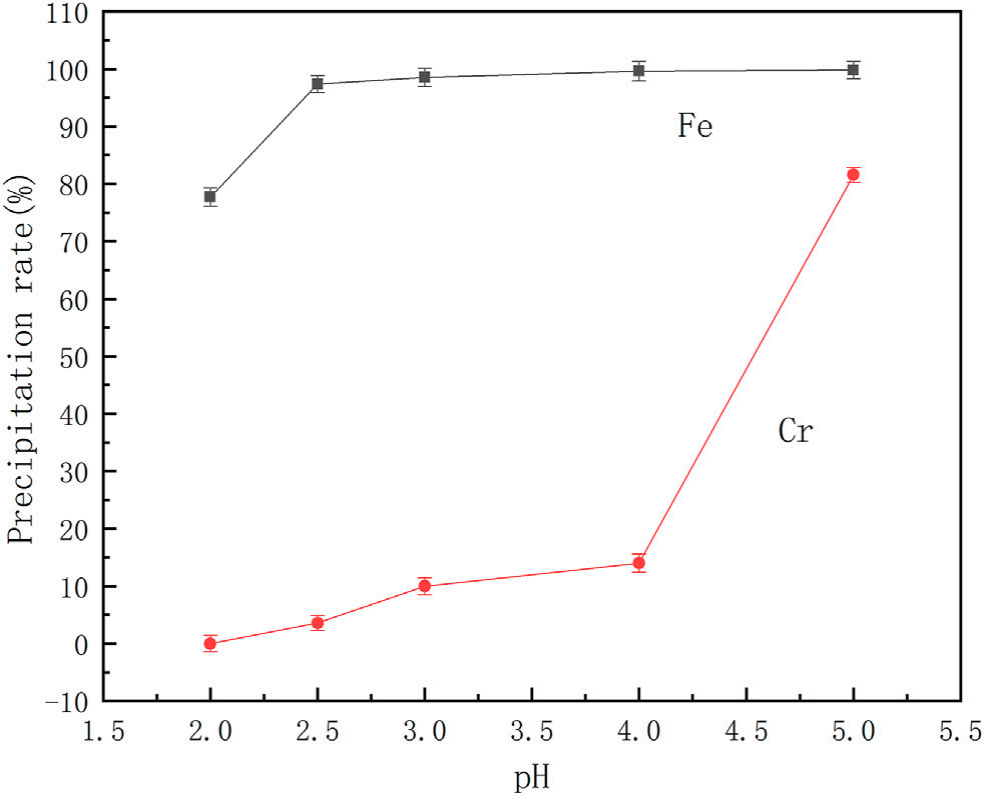
Effect of reaction pH on the precipitation rate of ferrochrome.

From the reaction Eqs. 1, 2 it can be shown that the coordination between benzoic acid and iron and chromium ions is affected by the pH of the solution. Therefore, a series of mixed ferrochromium solutions were prepared. After adding benzoic acid, the pH of the solution was adjusted to different values, and the effect of pH on the separation of ferrochromium was investigated. The results are shown in Figure 4.

It can be seen from Figure 4 that the precipitation rate of ferrochromium increases with an increase in pH. At a pH of 2.5, the rate of precipitation of Fe is 93.37%, and that of Cr only 3.61%. Following an increase in the pH value, the rate of precipitation of the iron did not change significantly. However, due to the hydrolysis of chromium ions, the rate of loss of Cr ions increased. Thus at a reaction pH of 2.5, chromium and iron ions can be effectively separated in the acid leaching solution of the electroplating sludge. At this point, the iron precipitation rate is 97.38% and the rate of loss of chromium is 3.59%.

#### Effect of Reaction Temperature on Ferrochrome Separation

**FIGURE 5:**
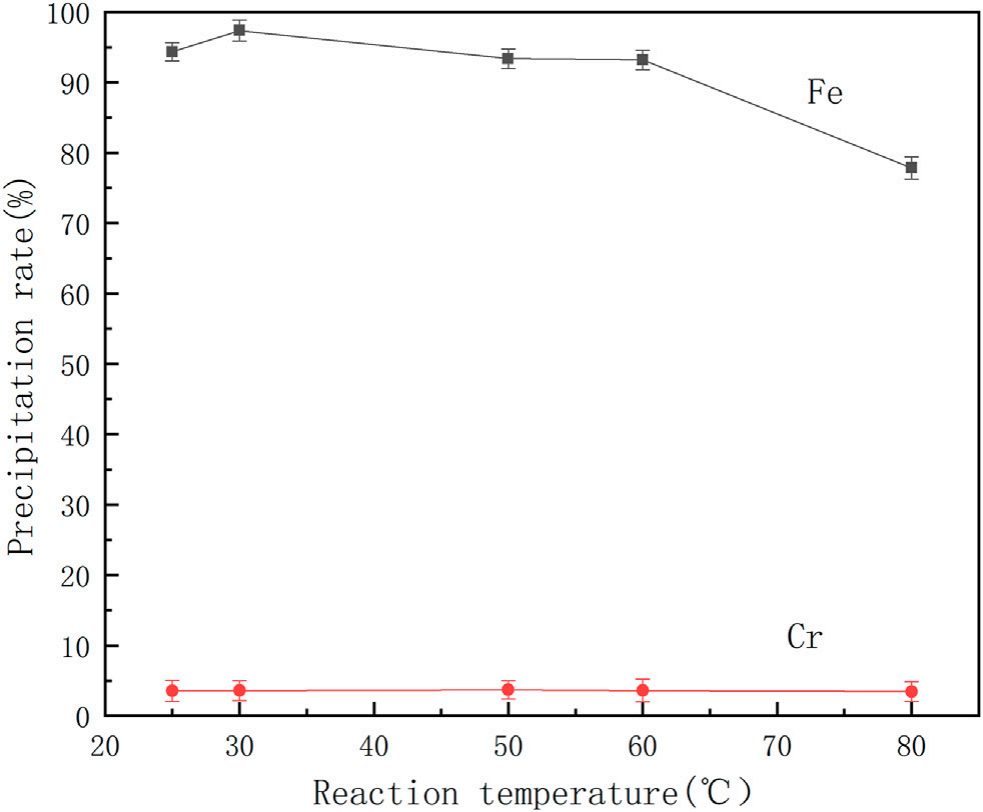
Effect of reaction temperature on precipitation rate of ferrochrome.

The coordination reaction between organic ligands and metal ions is easily affected by the temperature of the solution. Therefore, a series of mixed ferrochrome solutions were prepared, benzoic acid was added at different temperatures and the pH of the solution was adjusted to 2.5 for the reactions. The effect of temperature on the separation of ferrochrome is shown in Figure 5.

It can be seen from Figure 5 that the precipitation rate of chromium is extremely low and does not significantly change with increasing temperature. When the temperature exceeds 60°C, the precipitation rate of iron begins to decrease significantly, indicating that the high temperature is not conducive to the coordination of benzoic acid and iron ions. When the reaction temperature is controlled at 30°C, the chromium-iron ions can be effectively separated from the acid leaching solution of the electroplating sludge. At this temperature, the iron precipitation rate is 97.38% and the rate of loss of chromium is 3.59%.

#### Effect of Reaction Time on the Separation of Ferrochrome

**FIGURE 6:**
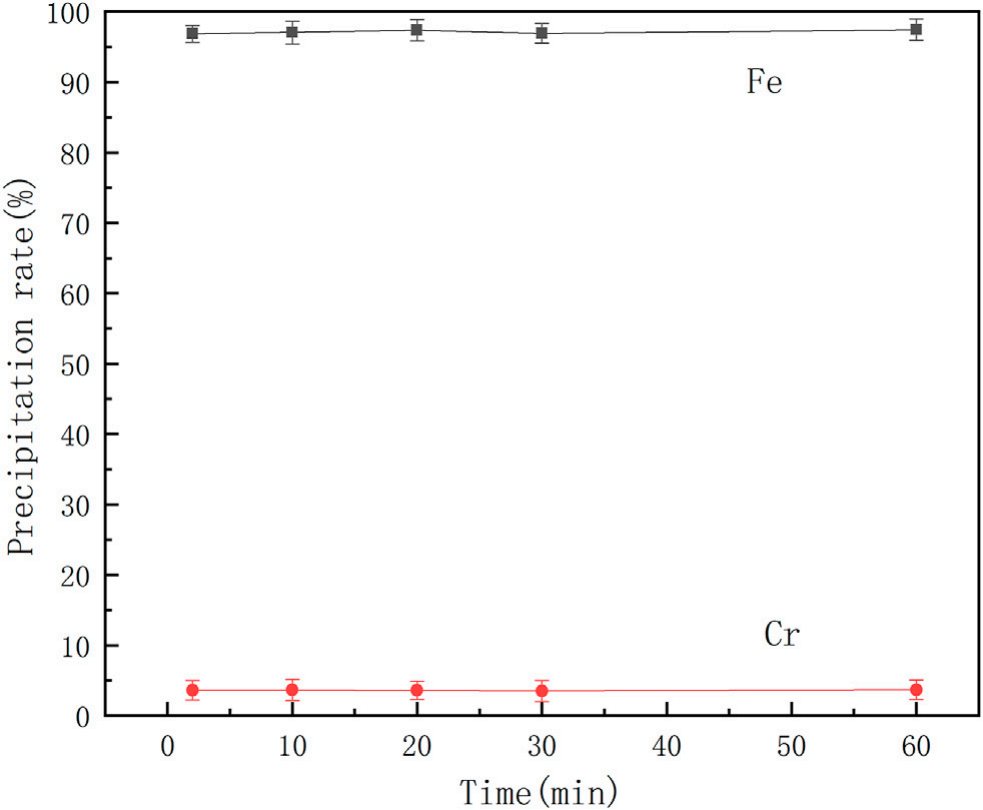
Effect of reaction time on separation of ferrochromium.

A series of mixed ferrochrome solutions were prepared, and reactions performed by adding benzoic acid and adjusting the solution pH to 2.5 at 30°C. The effect of the reaction time on the separation of ferrochrome was studied, and the results are shown in Figure 6.

It can be seen from Figure 6 that the precipitation rates of chromium and iron change little with time. The iron precipitation rate remains at almost 97%, while the chromium loss rate remains at 3.6%. Therefore, when the reaction time is controlled to approximately 2 min, chromium-iron ions can be effectively separated from the acid leaching solution of the electroplating sludge. At this time, the iron precipitation rate is 96.85% and the chromium loss rate is 3.62%.

#### Effect of Benzoic Acid Dosage on Separation of Ferrochrome

**FIGURE 7:**
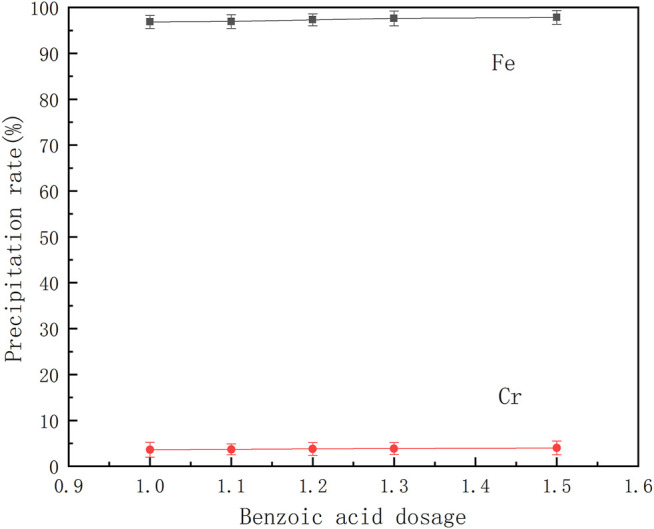
Effect of benzoic acid dosage on the separation of ferrochromium.

From Eqs. 1, 2, it can be seen that the coordination reaction between benzoic acid and iron and chromium ions is affected by the concentration of the reaction materials. Therefore, a series of mixed ferrochrome solutions were prepared. Different amounts of benzoic acid, for complete reaction with iron ions, were added at 30°C, and then the pH of the solution was adjusted to 2.5 to study the effects of the amount of benzoic acid on the separation of ferrochrome. The results are shown in Figure 7.

It can be seen from Figure 7 that the precipitation rates of chromium and iron both increase with an increase in benzoic acid dosage, but the increase is very small. An increased amount of benzoic acid does not achieve better separation of the ferrochromium. In conclusion, the ratio of benzoic acid to iron ions in the solution was adjusted to 3:1, so as to effectively separate chromium and iron ions in the acid leaching solution of the sludge.

#### Orthogonal Experiment

Based on the four parameters of reaction temperature, time, and solution pH, and according to the benzoic acid consumption and the precipitation rate of iron ions, three levels were designed using orthogonal experimental methods. The results are shown in Tables 1, 2, and 3.

**TABLE 1:** Orthogonal experiments and horizontal design.

Horizontal design	Factors			
	A Temperature (°C)	B Time (min)	C pH	D Benzoic acid dosage
1	30	2	2	1
2	50	20	2.5	1.2
3	70	60	3	1.5

**TABLE 2:** Results of the orthogonal test and range analysis L_9_ (3^4^).

Order number	A Temperature (°C)	B Time (min)	C pH	D Benzoic acid dosage	Iron precipitation rate (%)
1	1(30)	1(2)	1(2)	1(1)	67.28
2	1(30)	2(20)	2(2.5)	2(1.2)	96.86
3	1(30)	3(60)	3(3)	3(1.5)	99.35
4	2(50)	1(2)	2(2.5)	3(1.5)	97.93
5	2(50)	2(20)	3(3)	1(1)	98.56
6	2(50)	3(60)	1(2)	2(1.2)	86.32
7	3(70)	1(2)	3(3)	2(1.2)	97.66
8	3(70)	2(20)	1(2)	3(1.5)	83.58
9	3(70)	3(60)	2(2.5)	1(1)	83.60
K1	87.830	87.623	79.060	83.147	
K2	94.270	93.000	92.797	93.613	
K3	88.280	89.757	98.523	93.620	
R	6.440	5.377	19.463	10.473	

**TABLE 3:** Variance results of orthogonal experiment.

Factors	The sum of variances	Degree of freedom	F Values	F critical-values
Temperature	77.556	2	0.330	4.460
Time	43.979	2	0.187	4.460
pH	600.312	2	2.552	4.460
Benzoic acid dosage	219.242	2	0.932	4.460
Error	941.09	8		

It can be seen from Tables 2, 3 that according to the selected factors and horizontal analysis, the order of the influence of each factor on the separating effect of ferrochrome is: C > D > A > B, that is, solution pH > benzoic acid dosage > reaction temperature > reaction time. The pH of the solution had the greatest effect on the separation of ferrochrome, and the reaction time had the least effect on the separation of ferrochrome. Therefore, the effect of solution pH on the rate of the precipitation of iron ions should be considered to be the major factor for the separation of benzoic acid complexed chromium iron.

In summary, benzoic acid was used as a complexing precipitant to separate Cr^3+^ and Fe^3+^ in the solution. The benzoic acid was added in a 1:3 molar ratio of benzoic acid: Fe^3+^and the temperature maintained at 30°C. The pH of the solution was adjusted to 2.5 with ammonia water and stirred for 2 min. During this period of time, the rate of precipitation of the iron reaches 96.85%, and the rate of loss of chromium is only 3.62%.

### Study on the Separation Mechanism of the Complexation Precipitation

#### Apparent Morphology Analysis

**FIGURE 8:**
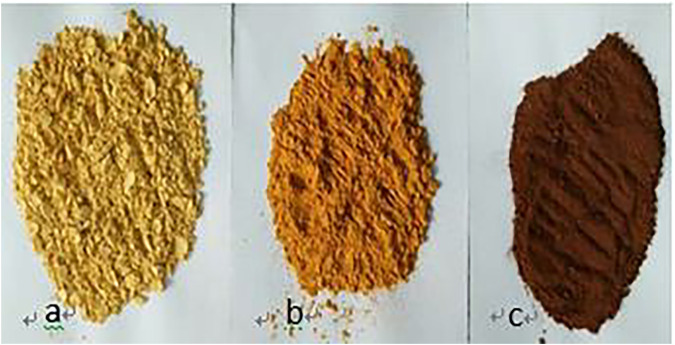
**A)** Separation of ferrochrome products by complexing precipitation at 30°C; **(B)** Separation of ferrochrome products by complexing precipitation at 50°C; **(C)** Separation of ferrochrome product by complexing precipitation at 80°C.

The benzoic acid complex precipitation products and separation of ferrochrome at different temperatures was conducted using different experiments. The actual products are shown in Figure 8.

It can be seen from Figure 8 that as the temperature increases, the appearance of the benzoic acid and ferrochrome complex changes, and its apparent color changes from cinnamon to dark-red, and gradually deepens. As the conditions of each experiment were the same except for temperature, it was speculated that the complexation mechanism of benzoic acid and ferrochrome is different at different temperatures. The mechanism cannot be determined from a change in apparent shape. Therefore, further analysis on the characteristics of the material structure of the separated products of the ferrochrome was conducted.

#### X-Ray Fluorescence Analysis

X-ray fluorescence analysis was used to detect the elements in the separated products of ferrochromium at different temperatures, as shown in Figure 8 and the test results are shown in Table 4.

**TABLE 4:** The elements in the separated products of the ferrochromium at different temperatures.

	Element content/%				
	O	Fe	Cr	S	Other
Separated product at 30°C	9.535	36.132	0.017	0.147	54.169
Separated product at 50°C	9.838	41.078	0.211	0.148	48.725
Separated product at 80°C	12.037	41.809	0.325	2.784	43.045

From the data in Table 4, it can be seen that from 30 to 50°C, the iron content increases while the chromium content remains almost unchanged. It can be concluded that within this temperature range, the rate of precipitation of iron increases, and the loss of chromium remains mostly unchanged. When the temperature rises from 50 to 80°C, the ferrochromium content remains almost unchanged. Therefore, in this temperature range, the rate of chromium precipitation increases, while the rate loss of iron is mostly unchanged.

#### Infrared Spectroscopic Analysis

**FIGURE 9:**
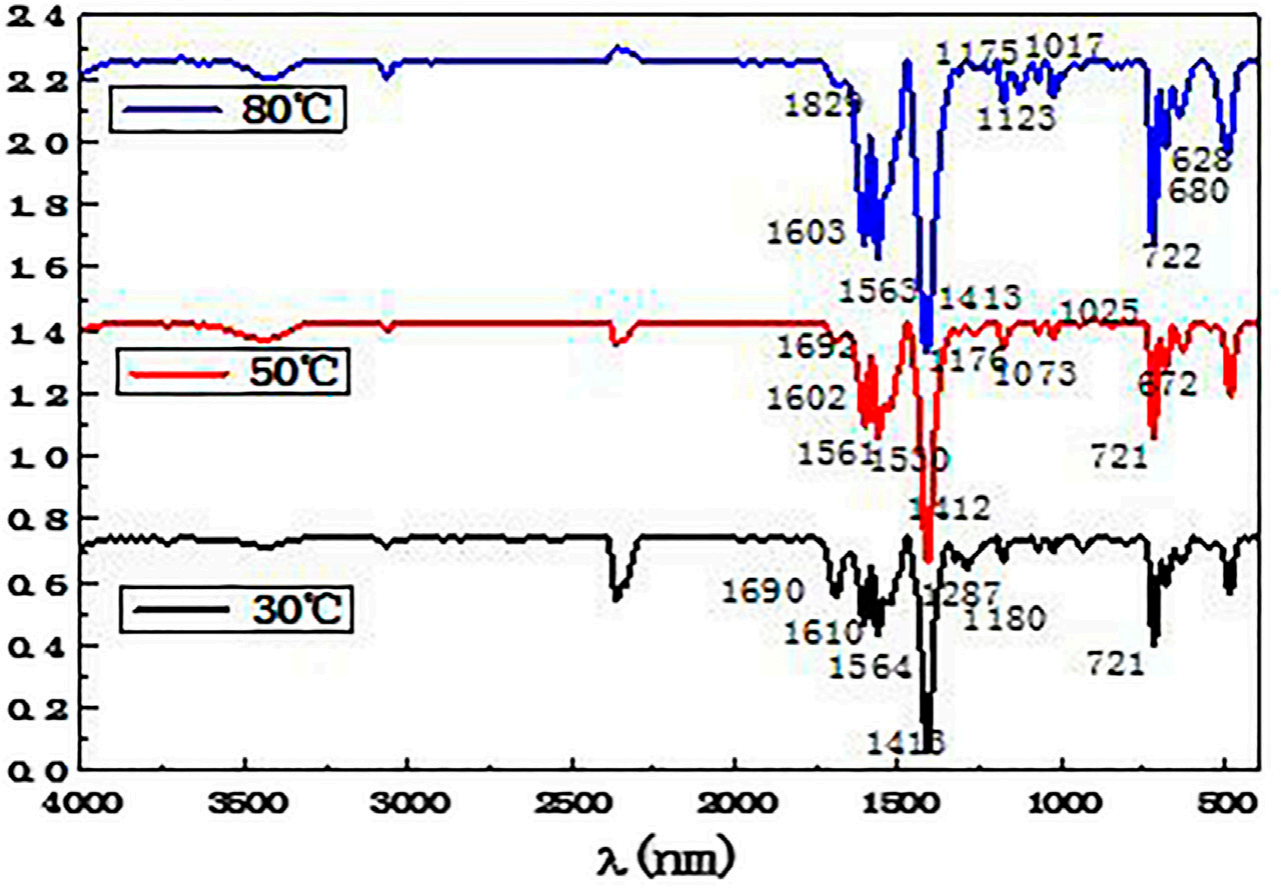
Infrared spectrogram of the product of the benzoic acid and ferrochrome mixed reaction.

Combined with Table 4, the elements present in the separated ferrochrome products at different temperatures were analyzed by infrared spectroscopy. The results are shown in Figure 9.

From the analysis in Figure 9, it can be seen that the absorption peaks appearing at approximately 1,601 and 1315cm ^−1^ in the three infrared spectra obtained at 30, 50, and 80°C correspond to the stretching vibration absorption bands of the benzene ring carbon bonds. The three characteristic absorption peaks of the benzene ring can be seen at 3,061, 1,601, and 721 cm^−1^. The strong absorption peaks at 1,564 and 1413 cm^−1^ can be ascribed to the asymmetrical and symmetrical stretching vibrations of the carboxylate ion in the benzoate. In all the spectra, strong absorption peaks can be observed at 636 and 492 cm^−1^, corresponding to iron benzoate. This proves that there are benzoic acid molecules in the products resulting from the precipitation and coordination reaction between benzoic acid and the iron ions. Combined with the results of the X-ray fluorescence analysis, it can be seen that the precipitated and separated product contains a significant number of iron and oxygen elements, which shows that the complexation of iron with benzoic acid is significant, whereas the amount of complexation with the chromium is small. Thus, selective complexation and separation of ferrochromium in the acid leaching solution of the electroplating sludge has been successfully achieved.

#### H NMR Spectral Analysis

**FIGURE 10:**
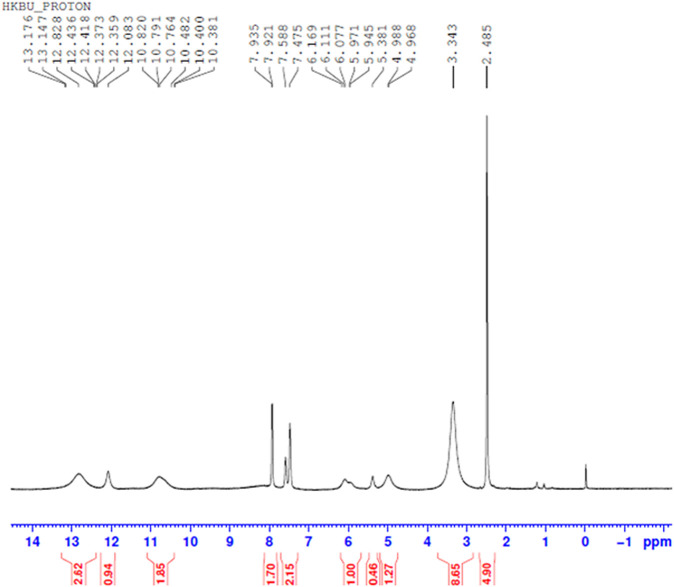
^1^H NMR spectrum resulting from the addition of benzoic acid to ferric sulfate solution at 30°C to obtain precipitated product.

**FIGURE 11:**
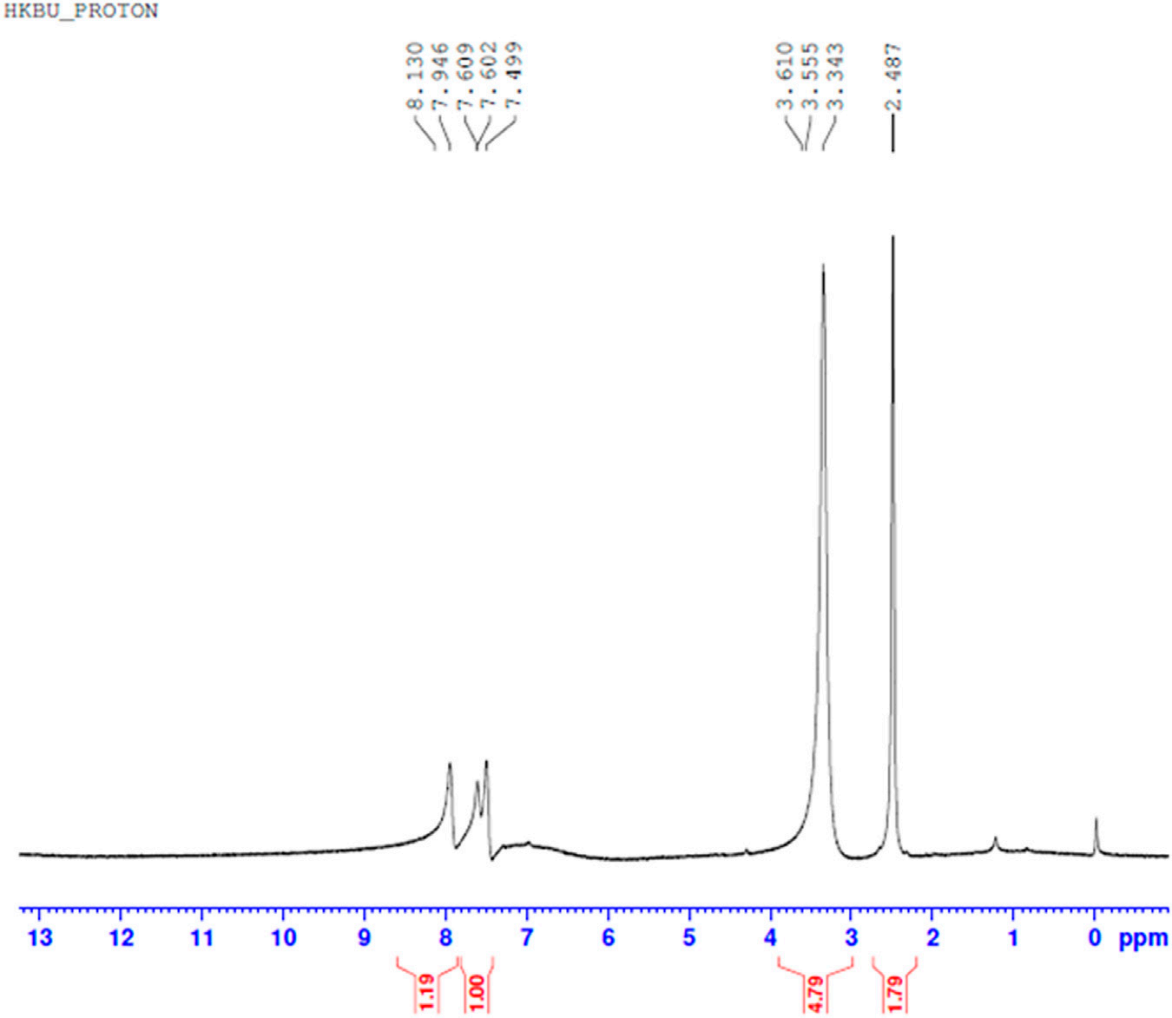
^1^H NMR spectrum resulting from the addition of benzoic acid to chromium sulfate solution at 30°C to obtain precipitated product.

**FIGURE 12:**
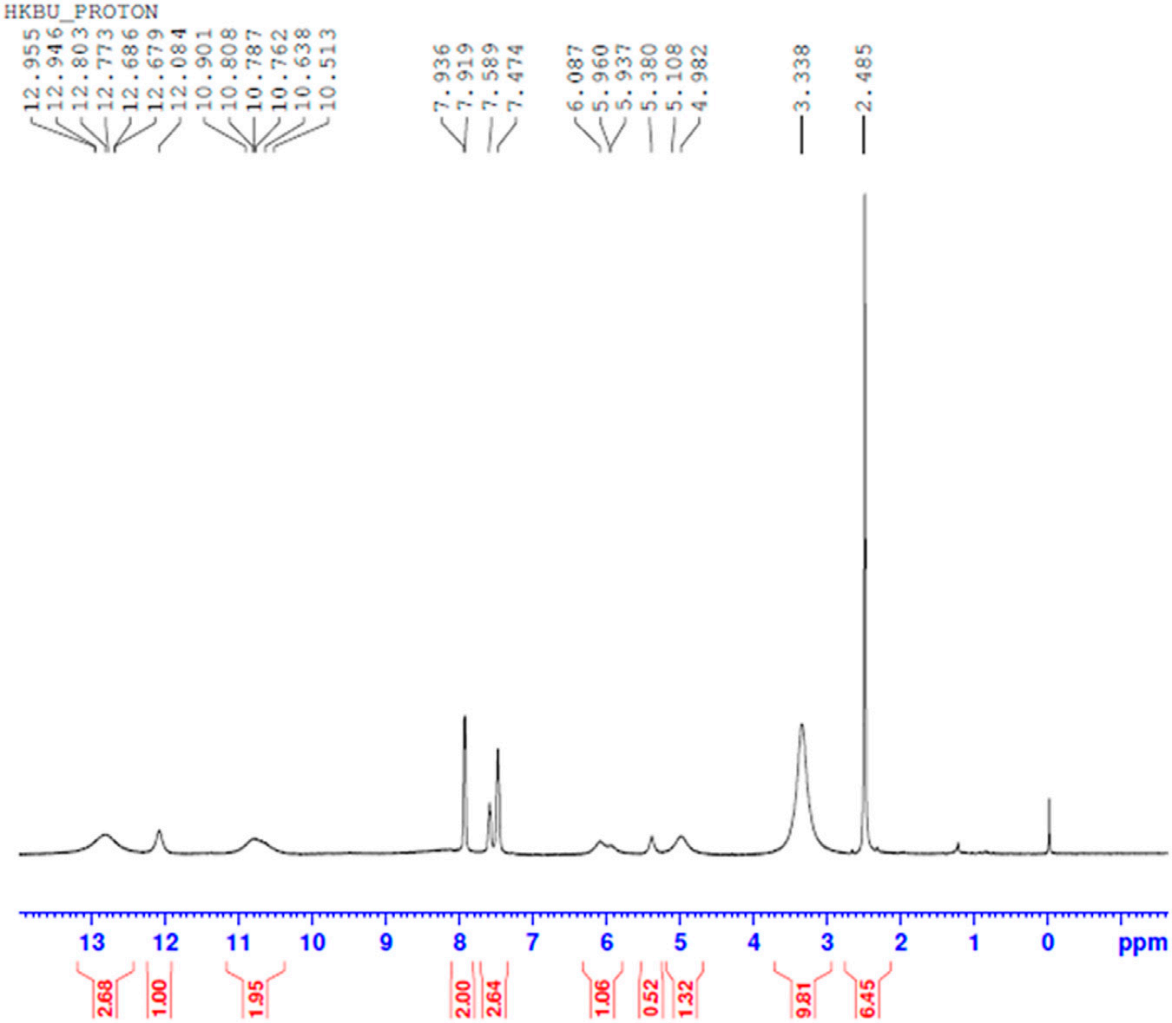
^1^H NMR spectrum resulting from the addition of benzoic acid to ferric sulfate and chromium sulfate mixed solution at 30°C.

**FIGURE 13:**
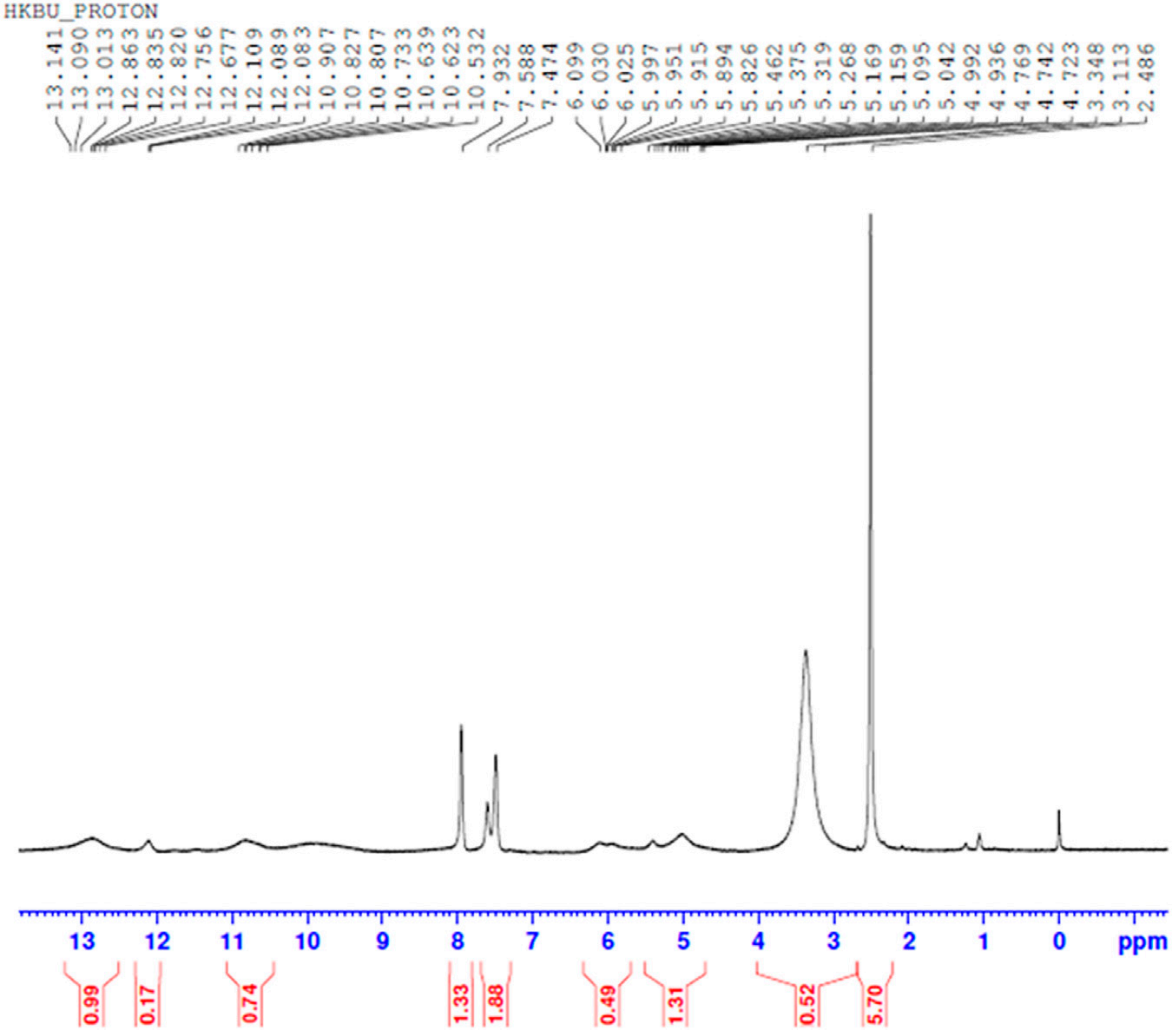
^1^H NMR spectrum resulting from the addition of benzoic acid to iron sulfate and chromium sulfate mixed solution at 80°C to obtain precipitated product.

Comparing Figures 10–13 H NMR spectrum, it can be seen that in the H NMR spectra of the reaction products obtained from the mixing of ferric sulfate and chromium sulfate with benzoic acid, the signals are in agreement with those of the reaction products of ferric sulfate and benzoic acid. The ability of ferric sulfate to react with benzoic acid is stronger than the ability of chromium sulfate to react with benzoic acid. This characteristic of benzoic acid can be used to achieve separation of chromium and iron from a mixed system (Note: DMSO was used as the solvent to dissolve the samples for testing).

## Conclusion

(1) An operationally simple procedure for the separation and precipitation of Cr^3+^ and Fe^3+^ using benzoic acid as a complexing agent and adjustment of the reaction solution pH with ammonia, has been developed. By adding benzoic acid in a 1:3 molar ratio of Fe^3+^: C_6_H_5_COOH, controlling the temperature at 30°C, adjusting the pH of the solution to 2.5 with ammonia, and stirring for 2 min, iron could be precipitated at a rate of 96.85% with only a 3.62% rate loss of chromium. The chromium and iron concentrations in the precipitated solution are 0.063 and 1.790 g/L, respectively.
(2) After using benzoic acid as a complexing agent, the crystal form of the precipitate changes. The precipitated iron benzoate particles are finer, the settlement rate is rapid, and the product is easy to filter. The problem associated with the filtration of colloidal ferric hydroxide produced by precipitation and neutralization of chromium and iron ions is avoided. The separation of Cr^3+^/Fe^3+^ from the acid leaching solution of electroplating sludge has been successfully achieved and the developed procedure will help promote further research in this area.

## Data Availability

The original contributions presented in the study are included in the article/Supplementary Material, further inquiries can be directed to the corresponding authors.
